# Feasibility and Acceptability of Tele-Colposcopy on the Caribbean Coast of Nicaragua: A Descriptive Mixed-Methods Study

**DOI:** 10.1089/tmr.2021.0024

**Published:** 2021-11-05

**Authors:** Emma McKim Mitchell, Aubrey L. Doede, Michelet McLean Estrada, Orlando Benito Granera, Francisco Maldonado, Brian Dunn, Shernai Banks, Imani Marks-Symeonides, Danielle Morrone, Charlotte Pitt, Rebecca A. Dillingham

**Affiliations:** ^1^ University of Virginia School of Nursing, Charlottesville, Virginia, USA.; ^2^ Fundacion Movicancer, Managua, Nicaragua.; ^3^ University of Virginia Karen S. Rheuban Center for Telehealth, Charlottesville, Virginia, USA.; ^4^ Main Line Health System, Paoli, Pennsylvania, USA.; ^5^ Bon Secours Health System, Richmond, Virginia, USA.; ^6^ University of Virginia Health System, Charlottesville, Virginia, USA.; ^7^ University of Virginia School of Medicine, University of Virginia Center for Global Health, Charlottesville, Virginia, USA.

**Keywords:** cervical cancer, mHealth, colposcopy, tele-colposcopy, provider perspectives, Telemedicine

## Abstract

**Background:** Cervical cancer, a preventable cancer of disparities, is the primary cause of cancer death for women in Nicaragua. Clinics and personnel in rural and remote Nicaragua may not be accessible to perform recommended screening or follow-up services.

**Objective:** To assess acceptability and feasibility of integrating innovations for high-quality screening and treatment follow-up (tele-colposcopy) into existing pathways on Nicaragua's Caribbean Coast within the context of the National Cervical Cancer Control Program.

**Methods:** Provider focus groups, key informant interviews, and environmental scans were conducted for 13 clinics on the Caribbean Coast of Nicaragua. Topics discussed included a smartphone-based mobile colposcope (MobileODT hardware and mobile platform), mobile connectivity capacity, clinic resources, provider acceptability, and current diagnostic and clinical protocols. We tested device connectivity through image upload availability and real-time video connection and simulated clinical encounters utilizing MobileODT and a low-cost cervical simulator. We developed a database of colposcopic images to establish feasibility of integrating this database and clinical characteristics into the cervical cancer registry.

**Results:** Provider acceptability of integrating tele-colposcopy into existing cancer control efforts was high. Image upload connectivity varied by location (mean = 1 h 9 min). Most clinics had running water (84.6%) and consistent electricity (92.3%), but some did not have access to landline telephones (53.8%).

**Conclusions:** As faster connectivity becomes available in remote settings, Mobile Health tools such as tele-colposcopy will be increasingly feasible to provide access to high-quality cervical cancer follow-up. World Health Organization guidance on integrating technology into existing programs will remain important to ensure programmatic efficacy, local relevance, and sustainability.

## Introduction

The World Health Organization (WHO) has adopted an elimination strategy for cervical cancer within the next 10 years, which defines specific vaccination, screening/early detection, and treatment targets to be achieved by 2030.^1^ For primary screening to result in reductions in cervical cancer incidence and mortality, patients must complete follow-up testing, re-screens at set intervals,^2,3^ and treatment when indicated.

### Cervical cancer control in Nicaragua

Roughly 85% of cervical cancer mortality is in low- and middle-income countries.^4^ Despite technological advances in cervical cancer screening, decreased access to health care has impacted cervical cancer control globally.^5^ In Nicaragua, where cervical cancer is the number one cancer killer of women,^6^ disparities in accessing prevention services persist and are growing.^2^ Although the human papillomavirus (HPV) vaccine is available for purchase,^7^ there is not currently a national vaccination program in Nicaragua.^2^ Intra-country variability in screening coverage is significant depending on geographic region; nearly 79% of women living in the Pacific region of Nicaragua have had a lifetime cervical screen, compared with 59% on the Caribbean Coast.^6^

A recent study^8^ involving scaling-up HPV-based primary screening in the Pacific and Central regions of Nicaragua screened 44,635 women for 4 years; however, significant loss-to-follow-up (nearly 72%) and the absence of women from the Caribbean Coast were noted.

**FIG. 1.**
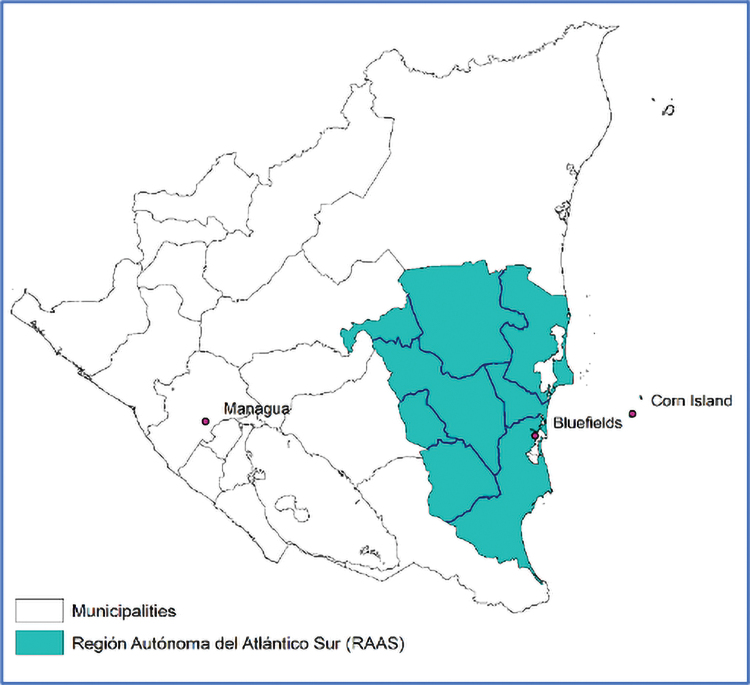
Catchment area for Bluefields MINSA/SILAIS, geographic target highlighted. MINSA, Nicaragua Ministry of Health; SILAIS, Sistemas Local de Atención Integral de La Salud.

Bluefields is the political seat and largest city on the Caribbean Coast of Nicaragua (Fig. 1) and home to the region's hospital.^8–12^ Barriers to cervical cancer control persist in this region. In Bluefields, screening services are provided at no cost through primary care clinics or at the region's hospital. For rural surrounding communities, screening services are provided at no cost through brigades, where health care providers travel to communities and return if follow-up is required.

To increase access to screening and early detection, and to provide ongoing quality improvement to the cervical cancer control program, the Nicaragua Ministry of Health (MINSA), in partnership with Fundación Movicáncer,^13^ developed Sistema de Vigilancia para la Prevención del Cáncer en la Mujer (SIVIPCAN), the national cervical and breast cancer surveillance system.^13^ Internationally recognized,^14^ SIVIPCAN has standardized data collection nationally, significantly impacting cervical cancer control.^15,16^ It has become an exemplar for cervical cancer registries in Latin America and the Caribbean.^14^

Upon a positive screening result (Pap testing and/or visual inspection with acetic acid [VIA] are standards of care), women in Nicaragua are commonly referred for cryotherapy,^8^ which allows for rapid treatment, but has a lower 6-month success rate in preventing recurrence of cervical lesions than other treatments.^17^

Referral for colposcopy before treatment is the gold standard for follow-up screening and diagnosis after a positive result,^18,19^ as it produces improved long-term outcomes with reduced recurrence.^17^ Colposcopy has a high sensitivity and specificity but is resource-intensive,^20^ as it requires a colposcope and a trained provider and may have significant inter-provider interpretation variability.^21^ Although referral for colposcopy after a positive Pap test is currently in the cervical cancer control algorithm,^14^ the Caribbean Coast of Nicaragua has a single colposcope, located in the city of Bluefields. In Nicaragua, follow-up is commonly performed using VIA,^2^ which remains a primary screening and diagnostic method for cervical abnormalities in low-resource settings, as it is less resource-intensive than colposcopy or cytology (Pap testing).^22,23^

### Mobile Health in cervical cancer control

Mobile Health (mHealth) has been used to augment in-person health care to scale up access and has particular relevance in rural and remote settings.^24^ MobileODT (MobileODT, Ltd., Tel Aviv0Yafo, Israel) has developed a mobile smartphone colposcope, paired with a cloud-based platform, to increase access to remote, reduced-cost screening by skilled providers.^25,26^ The device costs considerably less than traditional equipment (∼$4,200 vs. $14,000).^27^ Its small size, long battery life,^28^ and low computational power use allow for transportation between patients and clinics, and the apparatus itself does not make physical contact with patients.^29^ Unlike conventional colposcopy, the MobileODT platform provides validated image capture,^29^ documentation, and comparison of VIA-treated cervical images. Images can be assessed remotely by expert practitioners through the enhanced visual assessment (EVA) cloud portal.^30^ Challenges remain for follow-up screening and treatment after a positive screening, particularly in rural and remote areas. Innovative low-cost technology has the potential to provide faster access to screening and treatment.

The purpose of this study was to explore the feasibility and acceptability of implementing these innovative strategies into the existing cervical cancer control program in Nicaragua to increase access to high-quality follow-up treatment upon a positive result while targeting a region where access remains challenging. The cervical cancer focus stems from a decade-long research collaboration between the University of Virginia (UVA) and several long-term in-country partners.^9–12^

## Materials and Methods

The WHO has provided a toolkit for the collection of cervical cancer control data, including guidance for capturing digital cervicography data and images.^31^ We assessed the acceptability and feasibility of incorporating tele-colposcopy using the MobileODT device and EVA cloud-based platform into existing efforts using these guidelines through provider focus groups, environmental scans for cellular connectivity and clinic resources, and a feasibility assessment of incorporating tele-colposcopy into Nicaragua's existing cervical cancer registry and control program. The UVA SBS-IRB approved this study and all participants provided informed consent.

### Phase 1: Provider perspectives on acceptability and feasibility

Using a descriptive mixed-methods approach, between December 2018 and March 2020, the study team traveled to Bluefields and surrounding communities five times to conduct key informant interviews and focus groups targeting health care providers and other stakeholders with relevant clinical experience. Initial inquiry centered on considerations for the continuum of cervical cancer control specific to this region, strengths and barriers of current services, systemic considerations, and provider-identified needs. We explored provider perspectives of potential mHealth approaches to increase access to cervical cancer control relevant to the region and target population, including tele-colposcopy using the MobileODT device and EVA platform.

**FIG. 2.**
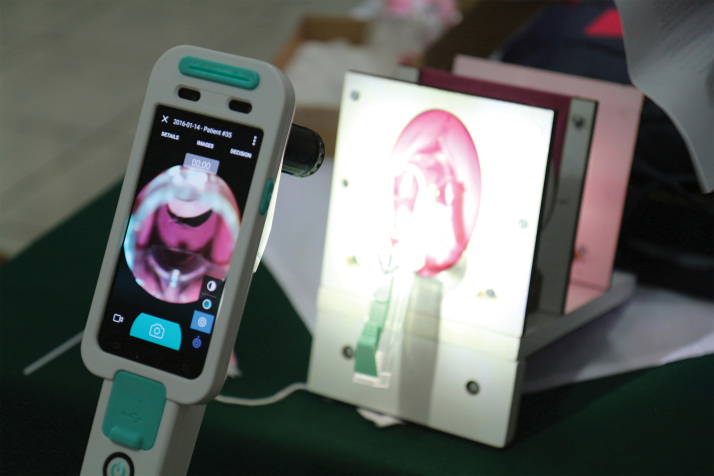
The LUCIA model consists of a pelvic frame, vaginal canal, and set of reusable cervical models to simulate variations of normal and abnormal clinical findings. Cervical models include four variations: cysts, cancer, acetowhite changes, and vascular changes.^30^ LUCIA, low-cost universal cervical cancer instructional apparatus.

To demonstrate this device using a human proxy, we employed the low-cost universal cervical cancer instructional apparatus (LUCIA), which has been developed for the purposes of training health care providers for cervical cancer screening and treatment skill development (Fig. 2).^32^ The LUCIA model was used in this study to demonstrate the MobileODT device and EVA cloud-based platform to health care providers, and subsequently to assess integration of images into SIVIPCAN.

#### Provider focus groups and key informant interviews

Three focus groups and two key informant interviews were conducted by the first and third authors during December, 2018, with nurses (*n* = 6) and physicians (*n* = 3 general practitioners, and *n* = 3 specialty providers) in Bluefields and surrounding rural communities, using an interview/focus group topic guide, to explore provider acceptability of integrating tele-colposcopy into existing cervical cancer control efforts. Participants were contacted through snowball sampling^33^ based on their roles in the health care system. Discussion topics included the MobileODT hardware and mobile platform, clinics' capacity for mobile connectivity, provider acceptability, current protocols surrounding diagnostics and patient care, the current SIVIPCAN database and intake screening form, and ability to contact patients for follow-up.

Interview discussions were audio-recorded and transcribed verbatim in the language they were conducted (English or Spanish).^34^ All transcripts were verified by one researcher for accuracy with a 10% subset evaluated by a second researcher for agreement. Using thematic analysis,^35^ transcripts were independently coded by two researchers in their original language to identify emergent themes and reach consensus. Four additional meetings took place with local health care leadership (*n* = 11) and key informants (*n* = 3). For these meetings we used the same interview/field note topic guide, and where audio recording was not possible, field notes were maintained and incorporated into the analysis.

### Phase 2: Environmental scan for feasibility of integration

Guided by the WHO Toolkit for Cervical Cancer Prevention,^31^ researchers explored specific feasibility parameters for potential implementation of tele-colposcopy in Bluefields and surrounding rural communities. These included cellular connectivity and individual clinic resources of access to clean running water, electricity, sanitation, and transport.^31^

#### Cellular connectivity

To explore the feasibility of utilizing tele-colposcopy to connect expert colposcopists with health care providers with limited colposcopic training, we assessed technical specifications necessary to deploy tele-colposcopy in the geographic target region. This involved measuring cellular connectivity at 13 public and private clinics in Bluefields and surrounding rural areas, assessing video and audio transmission quality, and establishing the feasibility of integrating cervical images captured through the MobileODT device and EVA platform into the SIVIPCAN national cervical cancer registry.

To test device connectivity, we captured images of human proxies. Citrus fruits were selected, as the skin texture provided visual variability similar to the cervical surface. Time between image capture and upload availability on the MobileODT EVA platform was documented. We assessed real-time connections while one study member was in Bluefields and surrounding communities and another in Virginia. Cisco Jabber, the standard Health Insurance Portability and Accountability Act-compliant platform used by the UVA Center for Telehealth,^36^ was employed to test real-time video connection in terms of delay of delivery (jitter) and loss of data (total packet loss), indicating the quality of real-time video connection as it would relate to a clinical encounter. These data were collected real time through analytics generated by the Cisco Jabber platform.

#### Clinic resources

To perform cervical screening and subsequent follow-up testing, WHO recommends access to clean running water, electricity, sanitation, and transport.^31^ In each clinic surveyed for the environmental scan (*n* = 13), we documented resources as present or absent using an adapted standardized documentation form for observations and structured interview questions for clinic staff from the WHO data collection toolkit^31^ upon its availability in January 2019.

### Phase 3: Integration of tele-colposcopy into SIVIPCAN

Using LUCIA (Fig. 2), we utilized the MobileODT device to capture 20 cervical pathology models, employing photo filters to capture three images of each model (normal, green, and red light), to simulate a clinical examination. We subsequently developed a database inserting these images into a spreadsheet to establish feasibility of uploading these photos and simulated intake forms into the SIVIPCAN registry. This was tested through a “data playground,” where SIVIPCAN was mirrored, preventing disruption of actual patient records.

## Results

### Phase 1: Provider perspectives on acceptability and feasibility

Through focus groups, key informant interviews, and demonstration of the MobileODT device utilizing LUCIA, providers and health care leaders expressed a high level of openness to integrating tele-colposcopy into current cervical cancer control efforts. Providers expressed equal desire to learn from patient perspectives regarding using this device for the procedure: “Everything is there. But sometimes the patient's fear is that you are going to have an image, and she is afraid of another person seeing it.” (Clinic Nurse, personal communication).

Providers and health care leaders discussed specific considerations for feasible implementation, including descriptions of the national cervical cancer control pathway and taking regional considerations into account. For example, rural and remote communities, for whom cervical cancer screening is mostly conducted through traveling brigades from Bluefields, could benefit from the access to a trained provider this device enables:

“Maybe we will not see her again…Most of the women will not go [because] they have to pay to travel to Bluefields, [but] they do not have the resources; they don't have money, and so our strategy [is] to be able to reach all of them with abnormal results…so that they can go to their follow-up until they are discharged. Because most want to go, but they cannot.” (Clinic Nurse, personal communication).

Cellular connectivity and variability in access to power were also potential barriers to implementation in rural and remote areas. The MobileODT device uses a rechargeable battery, and the cloud-based platform requires internet access to upload images and receive remote expert provider interpretation (although images and data entered can remain on the device until it next has cellular connectivity). A third resource discussed was the availability of pathologists. Pap tests are processed by pathologists in Bluefields.

Study participants were encouraged that the time interval between sample collection and interpretation (often 30–90 days) could be reduced significantly if point-of-care providers could connect with pathologists through this device, for interpretation. Furthermore, participants noted that shortening this time interval could reduce loss-to-follow-up: “The [cytology sample] that we take here, we send to the pathologist in Bluefields. We are sometimes delayed in this process at least a week to consolidate the registry and send the Pap…Bluefields takes more or less a month to return the results.” (Physician, personal communication).

Considerations specific to feasibility of deploying the MobileODT device included the strength and quality of images captured, including the photo filters allowing for comprehensive clinical examination. Although patients' discomfort with the speculum itself is one of many barriers participants discussed, the ease with which the MoblieODT device could be used with multiple patients was seen as a strength, as it does not contact patients.

### Phase 2: Environmental scan for feasibility of integration

Environmental scans included 13 health care sites. Only one site, a private clinic, provides HPV vaccination (for purchase). All sites currently perform Pap tests, and two have a colposcopist on staff. Whereas no site had wireless internet access (all clinics use cellular data), seven clinics (52.8%) currently have landline telephones, and all but one clinic has consistent power. Only two clinics did not have running water, and all clinics had access to basic sanitation services for patients, visitors, and staff. Within the city of Bluefields, transport is readily accessible through an inexpensive and extensive network of private taxis and a more limited system of public buses. Transportation becomes a significant barrier to accessing services for those living in the rural communities surrounding Bluefields. In some communities, waterways are the most reliable forms of transportation, with some distances requiring eight or more hours by boat to access health care services in Bluefields.

#### Connectivity findings

Of the 13 sites scanned for cellular connectivity, all involved photo uploads, and 9 involved video tests (Table 1), resulting in a mean of 4 photos (∼3 to 8, median = 3, standard deviation [SD] = 1.63) and 1–2 videos per site (mean 1.15, median = 1, SD = 0.376). The time interval varied considerably based on where an image or video upload took place. The time from photo upload to availability on MobileODT's cloud-based platform ranged from 7 min to 3 h 42 min (mean = 75.85 min, median = 15 min, SD = 87.82 min). When examining total packet loss (units for transmission and reception),^38^ there was a mean audio transmission loss of 4.87% (0.00–30.28%, median = 0%) and mean audio reception loss of 0.97% (0.00–3.28%). Regarding video data, the mean total packet loss was 12.64% (0.00–63.19%, median = 3.1, SD = 21.22) during transmission and 3.54% (0.00–14.11%, median = 3.15, SD = 4.54) when receiving data. For all video uploads, no pixilation was observed.

**Table 1.** Connectivity Data for Mobile ODT and Video Data for Jabber

Clinic	Number of photos captured	Number of photos + Videos at each location	Time from upload (photos and videos) to platform availability	maVideo transmission total packet loss (%, from Bluefields)	Video receiving total packet loss (%, from Charlottesville)	Sending jitter (milliseconds, from Bluefields)	Receiving jitter (milliseconds, from Charlottesville)
Private 1	8	10	0 h 37 min	63.19	0.00	14	32
Public 1	3	4	3 h 04 min	8.27	5.43	—	31
Public 2	4	5	0 h 15 min	—	—	27	—
Private 2	3	4	3 h 15 min	29.76	4.97	—	29
Public 3	3	4	0 h 10 min	0.00	0.15	20	12
Public 4	3	4	3 h 16 min	0.00	3.80	6	31
Public 5	3	4	1 h 14 min	9.22	3.15	29	44
Public 6	4	5	0 h 13 min	—	—	12	—
Public 7	3	4	0 h 12 min	0.26	0.00	—	11
Public 8	3	4	0 h 11 min	3.10	0.24	5	15
Public 9	6	7	0 h 07 min	—	—	16	—
Public 10	6	8	0 h 10 min	—	—	—	—
Public 11	3	4	3 h 42 min	0.00	14.11	25	8
**Mean (SD)**	**4.0 (1.63)**	**5.15 (1.95)**	**1 h 9 min**	—	—	—	—
**Median**	**3.0**	**4.0**					

Bold indicates mean, median and standard deviation of photos and videos captured.

“—” indicates data not captured.

SD, standard deviation.

### Phase 3: Integration of tele-colposcopy into SIVIPCAN

As SIVIPCAN utilizes a single intake form nationwide, simulating 20 records of hypothetical patients and their subsequent images (through LUCIA, Fig. 2) with a spreadsheet, enabled us to explore uploading to the “data playground.” Three images per patient (200 KB per image uploaded at 0.5 sec) were possible. We successfully uploaded images and simulated patient files into the “data playground,” indicating integration of tele-colposcopy would be possible in the SIVIPCAN system to inform clinical care.

## Discussion

mHealth-based tele-colposcopy on the Caribbean Coast of Nicaragua is acceptable to providers, feasible technologically, and can be integrated with the existing national registry. When exploring challenges in cervical cancer control, most providers noted their greatest concerns were for women in surrounding rural communities due to their lack of access and associated poor health outcomes. The ease of use and ability for providers (skilled or inexperienced in colposcopy) to capture and preserve images as part of a patient's health record had particular appeal for providers who worked in or traveled to those communities.

Several considerations for service delivery in other rural and remote settings should be addressed, including considering connectivity as quality indicators for program planning. Combining the MobileODT device with teaching and demonstration utilizing the LUCIA model^32^ allowed for practical exploration of technological capabilities in evaluating acceptability and feasibility of incorporating the device into existing cervical cancer control efforts. Being at a public or private clinic did not impact connectivity. Of note, connectivity times reflected herein are shifting: measures were captured before the Caribbean Coast transferred to 4G connectivity (rollout is ongoing). Therefore, shorter periods between image capture and availability on the EVA portal, as well as potentially improved video metrics, may evolve. This was the case in a parallel exploration of connectivity in Roatán, Honduras, which has 4G connectivity.^38^

Artificial intelligence (AI) and machine-based learning algorithms^39,40^ are being designed and clinically tested against different screening modalities.^41^ Clinical validation is necessary before scale-up efforts in resource-limited settings to determine efficacy and efficiency when compared with expert clinicians (colposcopists and other women's health care providers) in screening and early detection of cancerous lesions. These approaches often began with digital image capture for cervical lesions when compared with standard of care^42,43^ and have grown rapidly to include cell-phone-based digital colposcopy^43^ and testing of pocket-sized colposcopes.^41^

Image-capturing devices may have more or less regionally specific relevance depending on whether they require a constant power source^29^ and/or cellular connectivity,^44^ resources for implementation (such as whether a speculum is required),^45^ whether ongoing device maintenance is necessary, potential for data breaches, and provider buy-in.^32^ The clinical relevance of colposcopy includes providers' abilities to biopsy suspicious lesions. In the context of the Caribbean Coast, if colposcopy were to be increased through the mHealth model, pathologists tasked with interpreting Pap tests and biopsies in the current cervical cancer control model would also make follow-up recommendations to obstetrician-gynecologist providers. Specific considerations in these settings included the ability to maintain power to recharge the MobileODT device and the availability of expert providers. At this stage, systems-level solutions to the bottle-neck phenomenon of limited specialty providers will need to be addressed before AI algorithms are clinically validated and designed to replace expert clinicians. In some resource-limited settings, colposcopy is not recommended due to resource requirements. More research is needed to determine the resource availability and cost-effectiveness of integration of tele-colposcopy into existing cervical cancer control programs with unique attention to region-specific deployment and scale-up considerations.

### Limitations

Although the sample for this study did not include all health care providers or decision-makers in MINSA, it did represent a variety of clinicians in exploring acceptability and feasibility of implementation of tele-colposcopy in this unique setting. In addition, acceptability considerations include those of health care providers and local health care leaders. Notably absent are the perspectives of patients on acceptability of incorporation of the MobileODT device into patient care. Our findings have particular relevance to the regionally mediated needs of the Caribbean Coast of Nicaragua and are not generalizable to the rest of the country. Nicaragua as a whole has experienced stark economic impacts from a nationwide political upheaval in 2018 and now from the impacts of coronavirus disease 2019 (COVID-19) and an unprecedented 2020 hurricane season. The impacts on infrastructure, personnel, and supplies will be significant.

## Conclusion

On the Caribbean Coast of Nicaragua, the necessary components of feasibility and health care provider acceptability allow for the integration of an mHealth device and platform into existing cervical cancer control measures, in particular in clinics serving rural and remote communities. Although additional research is needed to determine other factors, such as cost-effectiveness and acceptability among patients, there is reason to suggest the possibility of mHealth integration into cancer control and screening access for this region of Nicaragua. Patient perspectives must be researched and integrated to promote program success. Furthermore, regionally tailored approaches will continue to be necessary to decrease barriers to cervical cancer preventive and treatment services, to reach vulnerable communities in support of the WHO's goal of cervical cancer elimination.^1^

## Abbreviations Used

AI: artificial intelligence
COVID-19: coronavirus disease 2019
EVA: enhanced visual assessment
LUCIA: low-cost universal cervical cancer instructional apparatus
MINSA: Nicaragua Ministry of Health
SD: standard deviation
SILAIS: Sistema Local de Atención Integral de La Salud
SIVIPCAN: Sistema de Vigilancia para la Prevención del Cáncer en la Mujer
UVA: University of Virginia
VIA: visual inspection with acetic acid
WHO: World Health Organization
